# Shared genetic influence on frailty and chronic widespread pain: a study from TwinsUK

**DOI:** 10.1093/ageing/afx122

**Published:** 2017-07-07

**Authors:** Gregory Livshits, Mary Ni Lochlainn, Ida Malkin, Ruth Bowyer, Serena Verdi, Claire J Steves, Frances M K Williams

**Affiliations:** 1 Department of Twin Research and Genetic Epidemiology, King’s College London, London, UK; 2 Department of Anatomy and Anthropology, Sackler Faculty of Medicine, Tel Aviv University, Tel Aviv, Israel; 3 Clinical Age Research Unit, King’s College Hospitals Foundation Trust, London, UK

**Keywords:** frailty index, fibromyalgia, twin, heritability, variance component analysis, older people

## Abstract

**Introduction:**

frailty is an increased vulnerability to adverse health outcomes, across multiple physiological systems, with both environmental and genetic drivers. The two most commonly used measures are Rockwood’s frailty index (FI) and Fried’s frailty phenotype (FP).

**Material and methods:**

the present study included 3626 individuals from the TwinsUK Adult Twin Registry. We used the classical twin model to determine whether FI and FP share the same latent aetiological factors. We also investigated the relationship between frailty and chronic widespread musculoskeletal pain (CWP), another holistic age-related condition with significant clinical impact.

**Results:**

FP and FI shared underlying genetic and environmental aetiology. CWP was associated with both frailty measures, and health deficits appeared to mediate the relationship between phenotypic frailty and pain. Latent genetic factors underpinning CWP were shared with frailty. While frailty was increased in the twins reporting pain, co-twin regression analysis indicated that the relationship between CWP and frailty is reduced after accounting for shared genetic and environmental factors.

**Conclusions:**

both measures of frailty tap the same root causes, thus this work helps unify frailty research. We confirmed a strong association between CWP and frailty, and showed a large and significant shared genetic aetiology of both phenomena. Our findings argue against pain being a significant causative factor in the development of frailty, favouring common causation. This study highlights the need to manage CWP in frail individuals and undertake a Comprehensive Geriatric Assessment in individuals presenting with CWP. Finally, the search for genetic factors underpinning CWP and frailty could be aided by integrating measures of pain and frailty.

## Introduction

The proportion of people older than 65 is growing faster than any other age group [1]. A systematic review in people over the age of 65 found a mean physical frailty prevalence of 9.9% (range: 4–17%), with higher prevalence when incorporating psychosocial frailty [2] and exponentially rising in older age groups [3]. With the world’s population aging, it has become imperative to clarify the underlying biological mechanisms that influence this process.

Frailty is defined as a state of increased vulnerability resulting from ageing-associated reduction in physiological reserve capacity across multiple systems, such that the ability to function in everyday life and respond to acute stressors is compromised. Frailty develops as a result of age-dependent deterioration in a variety of physiological functions and it leads to an increased risk of illness, dependency and adverse outcomes including falls, delirium and disability [4].

Two leading methods for identifying frailty exist, and both are predictive in terms of dependency and death. The first method, designed by Fried *et al*. [5] categorises frailty phenotype (FP) as a clinical syndrome using physical health measures. Limitations to this definition include the omission of disorders of cognition and mood [6] and its inability to stratify people without performance-based tests [7]. An alternative conceptualisation of frailty is as a multidimensional state quantifying the number, rather than detailing the nature of health problems. Rockwood’s frailty index (FI) model employs a well-defined method to create an index as a proportion of deficits [8, 9].

Much debate has taken place around whether these characterisations of the same theoretical phenomenon are directly comparable. It has been shown that the two methods show moderate correspondence to one another [10]. Nevertheless, there is a lingering question over the extent to which these different characterisations reflect the same underlying processes.

Chronic widespread musculoskeletal pain (CWP) is defined as pain lasting ≥3 months, located axially, above and below the waist, and on both sides of the body, with prevalence at 10–15% in the general population [11]. We and others have shown CWP to be heritable [12]. Recently a review paper highlighted a lack of prospective data to make inferences about the direction of the relationship between CWP and frailty [13]. Since then, the longitudinal studies have reported that CWP is associated with subsequent development of frailty [14–16]. This has been interpreted as a causal association; that pain states themselves decrease reserve and impact physiological systems, e.g. through perturbations in stress hormones, altered sleep and nutrition [17]. These studies classified frailty categorically. This phenomenon may have developed continuously over some time before crossing the threshold to be defined as frail. Therefore, despite the longitudinal nature of these studies, it is possible that common or reverse causation explain the association.

This study was designed to test to what extent these three associated phenomena, the FP, FI and CWP, share common aetiology, including possible contribution of the latent factors underlying a phenotype manifestation, which are a consequence of heritable factors, environmental factors shared by twin-pairs and non-shared (unique) environmental factors.

## Material and methods

### Sample

The data examined in the present study were from the TwinsUK Adult Twin Registry (described elsewhere: [18]). Participants were female volunteers who gave written informed consent approved by the St. Thomas’ Hospital research ethics committee. In total, 3,626 individuals (1,694 MZ, 1,158 DZ twins and 780 singletons) were assessed for both frailty measures. Information on CWP status was available on 1,770 participants (Table 1). The age ranged from 17 to 93 years, with mean 60.5 ± 13.9 years. Potential covariates, including age at menarche, smoking and body composition were obtained. Zygosity was established by standardised questionnaire, and zygosity confirmed by genotyping. Twins have been shown to be similar to age-matched singletons for a range of health and lifestyle variables [19].

**Table 1.** Basic descriptive statistics by zygosity of the study sample (*U*-test, Mann–Whitney *U* test; ANOVA, one-way analysis of variance using normalised and standardised data).

Variable	Category	Monozygotic twins		Dizygotic twins		Comparison
		Mean||%	SD	Mean||%	SD	*P*-value
Age (years)	N/A	58.44	15.15	63.49	11.13	4.56E-20 [*U*-test]
FI-score	N/A	−0.09	0.99	0.12	0.99	1.73E-10 [ANOVA]
FP-score	0	58.4%		57.3%		4.7E-01 [*χ*^2^, df = 3.0]
	1	29.3%		29.7%		
	2	9.9%		9.7%		
	>2	2.4%		3.2%		
CWP	Affected	18.4%		22.1%		5.5E-02 [*χ*^2^, df = 1]
	Not affected	81.6%		77.9%		
BMI (kg/m^2^)	N/A	25.77	14.44	26.57	14.93	7.3E-02 [ANOVA]
FBM (kg)	N/A	24.41	8.22	25.71	8.78	2.57E-04 [ANOVA]
SMM (kg)	N/A	17.37	2.55	17.88	2.83	1.2E-05 [ANOVA]
Smoking	Current smokers	7.8%	N/A	10.7%	N/A	1.38E-04 [*χ*^2^, df = 2)]
	Former smokers	27.9%	N/A	31.4%	N/A	
	Non smokers	64.3%	N/A	57.9%	N/A	

### Rockwood FI assessment

The FI was created as a proportion of deficits [20, 21] using data from the Healthy Ageing Twin Study [22]. In total, 33 domains of binary health deficit were created from questionnaire data and clinical tests covering a range of aspects of physiological and mental health, but excluding the pain domain. Each individual received a sum of deficits (units) from the entire list of questionnaires which was divided by the number of domains completed. FI behaved as a quantitative continuous trait, with a *γ* distribution (median: 0.19, IQR = 0.18).

### Fried FP assessment

As proposed by Fried *et al.* [5], the FP was based on examination of five physical characteristics; grip strength, timed walking, unintentional weight loss, fatigue and physical activity. Scores varied from 0 to 5, and were available in 3,257 individuals. While scores greater than three are classified as frailty, we analysed this trait as an ordinal variable in order to have maximal power.

### CWP assessment

The London Fibromyalgia Epidemiology Study Screening Questionnaire (LFESSQ) had been sent to twins for self-completion, without reference to the co-twin [23], and has been described by us previously. These participants were included in the CWP genome-wide association study meta-analysis and in the recent omics study [24].

### Smoking

The present sample included 3,622 individuals for whom the information on smoking habits was available. Of these, 2,230 (61.6%) individuals had never smoked 327 (9.0%) current smokers and 1,065 (29.4%) ex-smokers.

### Body composition

Basic anthropometrical measurements, including body weight (kg), height (cm), BMI (kg/m^2^) and measurements of body composition were taken at a clinical visit. Body composition components, including fat body mass (g) and lean body mass (g) were measured using the standard whole body dual-energy x-ray absorptiometry method [24]. Using calculation akin to that of BMI (kg/m^2^), we examined the effect of relative fat (FBM/*H*^2^) and lean body mass (LBM/*H*^2^).

### Statistical analysis

Basic descriptive statistics and association/correlation analysis between FI, FP and potential covariates (including age, smoking and body composition) was performed. Next, we implemented multiple logistic regression, with CWP as dependent variable, and age, smoking, body composition, FI/FP scores and CWP-status of co-twin as covariates. Smoking was not related to CWP and so was omitted from this analysis.

We further performed family-based variance decomposition analysis (Supplementary methods). This analysis is grounded on a classical quantitative-genetic theory that allows evaluation of the contribution of the additive genetic factors (*V*_AD_), common twin environment (*V*_TW_) and the residual component of variance (*V*_RS_) on the total inter-individual variation of the phenotype. The analysis could be extended to dichotomous phenotypes, e.g. affected versus non-affected [25], as implemented in MAN package [26]. This package allows bivariate analysis, which provides maximum likelihood estimates of the genetic (*R*_G_) and environmental (*R*_E_) correlations between the phenotypes of interest. These correlations reflect the extent to which genetic and environmental variation of the two phenotypes share common genetic and/or environmental effects. MAN allows calculations of *R*_G_ and *R*_E_ between the quantitative continuous variables and dichotomous phenotypes, with simultaneous adjustment for covariates.

## Results

### Descriptive statistics of FI and FP

The basic descriptive statistics of the study phenotypes, according to twin’s zygosity presented in Table 1. FI scores were root-square transformed to achieve normality and standardised (Fig S1, Supplementary material are available in *Age and Ageing* online). There were significant differences between the MZ and DZ twins, in particular with respect to age. The MZ twins appear to be younger than DZ twins. The age differences, however, explain most of the differences in other phenotypes. FI, for example, significantly correlated with age (*r* = 0.383, *P* < 0.0001, Fig S2, Supplementary data are available in *Age and Ageing* online), BMI (*r* = 0.276, *P* < 0.0001) and relative fat mass, FBM/*H*^2^ (*r* = 0.334, *P* < 0.0001). The FI scores increased with smoking status (0.0626 in never smokers, 0.2171 in ex-smokers and 0.2664 in current smokers, trend (*F* = 7.92, df = 2, *P* = 0.0004). The FP score similarly showed correlation with age (*r *= 0.186, *P* < 0.0001), BMI (*r* = 0.193, *P* < 0.0001) and FBM/*H*^2^ (*r* = 0.233, *P* < 0.0001). FI and FP scores were moderately correlated with one another (*r* = 0.536, *P* < 0.0001). As FBM/H^2^ was highly correlated with BMI (*r* = 0.831, *P* < 0.0001) and showed stronger associations with frailty, it was the measure of body composition taken forward in subsequent analyses.

### Relationship between FI and FP scores and CWP

Comparing the FI scores between the CWP affected (mean = 0.35, SD = 0.15, *N* = 357) and non-affected (mean = 0.20, SD = 0.10, *N* = 1413) individuals showed clear elevation in FI with CWP (*P* < 10^−10^). Simultaneous adjustment for covariates (ANCOVA) did not change the results of the comparison (*P* < 10^−8^). Multiple logistic regression analysis with CWP as dependent variable with age, FBM/*H*^2^ and FI as independent covariates, showed that FI and age, but not FBM/*H*^2^, showed significant independent association with CWP (Table 2). In addition this model takes into account the status of the co-twin and shows their highly significant association with CWP status.

**Table 2.** Contribution of risk factors for CWP including FI.

Covariate	Estimate	SE	*P*-value	OR (per SD/unit)	−95% CL	+95% CL
Age	2.7583	0.8246	8.4^−04^	15.772	3.1291	79.5038
AGE^2^	−2.7776	0.7533	2.3^−04^	0.0622	0.0142	0.2726
FBM/H^2^	−0.0166	0.0808	0.082	0.9835	0.8393	1.1525
FI_score	1.2950	0.1003	<10^−10^	3.6510	2.9987	4.4451
DZCWP	1.3258	0.2056	1.5^−10^	3.7652	2.5157	5.6352
MZCWP	1.6669	0.2191	4.8^−14^	5.2958	3.4459	8.1386

Multiple logistic regression analysis of CWP. The covariates tested in the analysis were age, age-squared (as the relationship with age was non-linear) FI-score, FBM/*H*^2^ and co-twin CWP-status.

The standardised FP scores were also significantly higher in CWP compared to unaffected individuals (0.85 ± 0.05 versus 0.49 ± 0.02, *P* < 0.001). This relationship remained unchanged after adjustment for covariates as above (Table 3).

**Table 3.** Contribution of risk factors for CWP including FP.

Covariate	Estimate	SE	*P*-value	OR (per SD/unit)	−95% CL	+95% CL
Age	0.1108	0.1019	0.2771	1.1172	0.9147	1.3644
FBM/*H*^2^	0.2394	0.0719	0.0009	1.2705	1.1034	1.4629
FP_score	0.2772	0.0673	4.04^−5^	1.3194	1.1562	1.5056
DZCWP	1.4686	0.1867	7.11^−15^	4.3433	3.0114	6.2642
MZCWP	1.9458	0.1990	6.57^−22^	6.9989	4.7372	10.3404

Multiple logistic regression analysis of CWP. The covariates tested in the analysis were age, age-squared (as the relationship with age appeared to be non-linear) FP-score, FBM/*H*^2^ and co-twin CWP-status.

### Heritability and co-heritability of FI and FP

Intraclass correlations for FI-scores were, for MZ and DZ twins 0.44, *P* < 0.001 and 0.25, *P* < 0.001 respectively, and for FP-scores 0.39, *P* < 0.001 and 0.22, *P* < 0.001 respectively. MZ correlations were significantly higher than DZ correlations (test for equality, *P* < 0.01), suggestive of genetic influence. We therefore undertook a series of univariate and bivariate analyses of the FI-, FP- and CWP-scores, with the main results summarised in Table 4.

**Table 4.** Estimation of the shared genetic and environmental factors to frailty and CWP.

Model parameters	Primary phenotypes, parameter estimates ± SE		
	CWP_score	FI_score	FP_score
*V*_AD_	0.661 ± 0.014	0.298 ± 0.029	0.250 ± 0.040
*V*_CE_	(F) 0	(F) 0	(F) 0
*V*_RS_	(C) 0.233	0.408 ± 0.024	0.692 ± 0.040
Covariates effect—regression parameters			
α	0.0038 ± 0.0029 ns	−0.196 ± 0.033	−0.1253 ± 0.0288
*β*__AGE_	0.166 ± 0.037	0.394 ± 0.040	0.227 ± 0.054
*β*__AGE_^2^	−0.092 ± 0.033	0.159 ± 0.0345	0.197 ± 0.046
*β*__FAT/H_^2^	0.183 ± 0.029	0.277 ± 0.021	0.312 ± 0.029
*β*__SMK_	0.114 ± 0.036	0.143 ± 0.031	(F) 0
*L*_F_	0.201	N/A	N/A
*L*_AB_	0.813	N/A	N/A
Pairwise correlations			
Type of correlation	CWP/FI_score	CWP/FP_score	FI_score/FP_score
*R*_AD_	0.638 ± 0.073	0.418 ± 0.108	0.570 ± 0.064
*R*_RS_	0.545 ± 0.072	0.055 ± 0.039 (NS)	0.443 ± 0.025
*R*_CE_	(*F*) 0	(*F*) 0	(*F*) 0

Variance component analysis of liability scores to CWP and FI and FP scores in the study sample. Variance components include: *V*_AD_—contribution of the additive genetic factors, *V*_TW_—contribution of the common family environmental factors shared by twins, *V*_RS_—contribution of the unknown (residual) factors. *L*_F_—prevalence of the condition in the sample, *L*_AB_—standardised (expressed in SDs) affection threshold of liability scores distribution. *R*_AD_, *R*_CE_, *R*_RS_—correlations between the genetic, common family environment and random environment effects respectively, (*F*) 0—parameter estimate statistically not significant, fixed to 0 without change in the likelihood of the model.

The upper part of the table provides parameter estimates from the corresponding most parsimonious univariate models. Despite the significant and independent effect of covariates age, relative fat body mass and smoking, the contribution of genetic factors to all three phenotypes was substantial and statistically significant. The heritability estimates varied from 25% for FP to 67% for CWP.

Highly significant genetic (0.570 ± 0.064) and environmental correlations (0.443 ± 0.025) were observed between the two frailty measures. Moreover, the results in the lower part of the table suggest that variation of frailty and CWP is largely governed by common genetic factors [for FI, genetic correlation (*R*_G_ = 0.638 ± 0.073) and FP, (*R*_G_ = 0.418 ± 0.108)] and in the case of the FI, also shared common environmental factors (*R*_E_ = 0.545 ± 0.072).

The partial genetic correlation between the CWP and FI remained high and significant (0.430, *P* < 0.001), when controlling for covariation in FP. However, the opposite combination of variables gave non-significant partial correlation, *r*(CWP, FP/FI) = −0.048 (*P *» 0.05). Finally, the correlation between the FI and FP scores (0.535, *P* < 0.001) remained virtually unchanged when CWP score variation was controlled. These results taken together indicate that FI and FP largely measure the same entity, and that FI is likely to be the ‘primary’ variable associated with CWP risk.

### Discordant MZ twin study

To test whether the association of CWP and FI is independent of these shared genetic and environmental factors we tested the difference in FI within monozygotic twin pairs discordant for CWP. Thirty five pairs were available, with mean FI score 0.326 (SD = 0.139) in the CWP affected versus 0.235 (SD = 0.110) in unaffected counterparts. These differences were statistically significant by pairwise *t*-test (3.37, *P* = 0.002) or by regular independent two samples *t*-test (3.04, *P* = 0.003). Remarkable, however, the level of frailty in the unaffected co-twin still trended higher than that observed in the total sample of CWP unaffected individuals (0.205, SD = 0.100), and in particular in pairs of both unaffected MZ twins (0.186, SD = 0.106). Using the twin-pair difference in FI and the twin-pair average together in the same logistic models as above, both these measures were independently predictive of CWP, but the effect size of the twin-pair average was significantly greater than the within-twin difference (*z* = 2.25, *P* = 0.025).

## Discussion

Using twin modelling we showed a significant heritability for both measures of frailty. Moreover, both genetic and environmental factors underpinning both characterisations of frailty were shared to a large extent. That is, both Fried and Rockwood’s approaches measure essentially the same underlying phenomena. Our data support previous reports of the heritability of frailty, suggesting, e.g. *h*^2^ = 0.43 (95% CI = 0.31−0.53) for FP in a sample of 3719 subjects in Denmark [27]). Recently our group reported a similar *h*^2^ = 0.45 (95% CI = 0.30−0.53) of the FI in an earlier phenotyping in the TwinsUK cohort [28]. The current study assessed the same cohort 5 years later, with data on both Fried’s FP and Rockwood’s FI in the same individuals at the same time. To our knowledge, no previous work has been able to measure the extent to which genetic and environmental factors are shared by these two measures. Our study indicates for the first time that FI and FP, to a large extent are tapping the same genetic and environmental sources of variance.

We found that both the FI and FP are associated with CWP, despite adjusting for known risk factors age and relative fat body mass. Analysis of the underlying determinants showed that variation in frailty and CWP was governed by common genetic factors and. in the case of FI, common environmental factors. Analysis indicated that the FI mediates the relationship between the FP and CWP, although this may be accounted for by the stronger statistical properties of a quantitative versus ordinal trait.

Discordant twin pair analysis and twin regression analysis are two methods which can test whether factors shared by twins explain the association between CWP and frailty. In twin pairs where one twin reports CWP but the other does not, frailty was greater in the CWP twin. This indicates that the association, while weaker, is still detectable given common genes and early environment. Regression analysis showed that within families, with the same genetic and early life background, the association between CWP and frailty was significantly weaker than between families, underlining that the association is likely to be partially explained by common causation.

The association between CWP and frailty has been evaluated in several population based studies, such as the European Male Ageing Study [14], and the English Longitudinal Study of Ageing [16], as well as in diseases states, including osteoarthritis [15] and chronic low back pain [29]. These studies have assessed frailty using the FI [7, 9] and FP [15, 29] approaches and all found evidence of association between frailty and CWP. Shega *et al.* [17] put forward the hypothesis that presence of persistent pain reduces physiological reserve and predisposes one to develop frailty. The longitudinal studies discussed above defined frailty using a threshold, and showed that CWP predicts change over that threshold. However, individuals with CWP may already have increased frailty, while not crossing the threshold to be defined as frail (reverse causation). Our study also showed that pain and frailty share some common underlying determinants, including a substantial genetic contribution (common causation). Exactly what these genetic factors are needs further investigation.

There are several limitations to this study. Firstly, the participants were all female, therefore the conclusions may not be applicable to men. It is known however that CWP is more prevalent in women (e.g. 30), as is frailty [3], and genotype–sex interaction in complex phenotypes is well recognised. As such, an all-female population in this setting may be considered advantageous.

Current standard Comprehensive Geriatric Assessment (CGA) does not include assessment for CWP. While pain may be addressed, typically in the setting of arthritis, there is no pain assessment included in battery of assessments [30]. The findings of this study highlights to clinicians the need to manage CWP in frail individuals, and to undertake a CGA in individuals with CWP. An international consensus group on frailty in 2013 recommended that all persons over age 70 be screened for frailty [3]. The findings of this study suggest that assessment for CWP should be included in such screening.

**Key points**

- Frailty measured using two separate ideological approaches shared underlying genetic and environmental aetiology.
- Chronic widespread pain associates with frailty, with health deficits mediating the relationship between frailty and pain.
- Latent genetic factors underpinning Chronic Widespread Pain are shared with frailty.

## Supplementary Material

Supplementary Data
