# Supplementary material for: Shared genetic influence on frailty and chronic widespread pain: a study from TwinsUK
Source: Age Ageing. 2017 Jul 7;47(1):119–25. doi: 10.1093/ageing/afx122 (PMC5860041; doi:10.1093/ageing/afx122)
Supplement: Supplementary Data [file aa-17-0169-file004.docx]

Gregory Livshits, Mary Ni Lochlainn, Ida Malkin, Ruth Bowyer, Serena Verdi, Claire J. Steves, Frances M. K. Williams. **Shared genetic influence on frailty and chronic widespread pain: a study from TwinsUK.** Age and Aging. 2017.

Supplementary Material

1. Family –based variance decomposition analysis (VCA).

To estimate the extent of the familial and possible genetic influences on interindividual variation of the quantitative continues phenotypes VCA was undertaken as implemented in statistical package MAN (Malkin and Ginzburg, 2015). The analysis is based on a quantitative genetic theory (Falconer and Mackay, 1996) and allows one to distinguish the different independent components forming the variation of the studied phenotype. This analysis uses a likelihood ratio test (LRT) of a series of hierarchically related models to evaluate the relative contribution of the additive genetic factors (V_AD_), common twin environment (V_TW_) and the residual component of variance (V_RS_) on the total inter-individual variation. Additionally, the model allows simultaneous estimation of the covariates (e.g. age, BMI etc) on the dependent variable. Heritability is a proportion of phenotypic variation attributable to putative genetic effects, i.e. h^2^= V_G_/V_Ph_. This program finds the best fitting and most parsimonious linear genetic model for the trait variability and produces maximum-likelihood estimates of the parameters on the basis of pedigree data.

When the study phenotypes (e.g. X and Y) correlate one may interested in the nature of the correlation. To distinguish between the genetic and environmental sources of covariation/correlation a bivariate variance component model could be implemented (Falconer and Mackay, 1996). The bivariate mixed model, as utilized in the MAN package calculates the variance components for each of the two phenotypes and evaluate an additive genetic [r_G(X,Y)_] as well as an environmental [r_E(X,Y)_] correlations between them. While genetic correlation is a quantitative measure of the shared effects of genes on each of the two traits under analysis, environmental correlation estimates the extent to which these traits share a common environment. As a regular correlation, both r_G_ and r_E_ may theoretically range between –1.0 and 1.0. The statistical significance of (r_G_) and (r_E_) was studied using nested models and by examining the change in chi-square values between the models.

As CWP is a dichotomous variable (affected vs non-affected) we implemented a well-established ‘quasi family-based variance decomposition analysis’ - QVCA (Malkin et al., 2014). This assumes the existence of an underlying liability to the disease (CWP) (Falconer 1965; Falconer and Mackay, 1996), and the existence of a liability threshold, τ (denoted later as L_Lab_), above which the individual is considered affected. Similara to VCA QVCA maximization procedure estimates the variance components in interindividual variation on liability scores(V**_AD_**, V**_CE_**, V**_RS_)** and the affection threshold τ on a liability scale. Furher deatials on this method are given elsewhere (Malkin et al., 2014).

**References**

Falconer DS. The inheritance of liability to certain diseases, estimated from the incidence among relatives. Ann Hum Genet [Internet]. 1965;29(1):51–76. Available from: <http://dx.doi.org/10.1111/j.1469-1809.1965.tb00500.x>

Falconer DS, Mackay TF. Introduction to Quantitive Genetics [Internet]. 4th ed. Harlow, Essex, UK: Longmans Green; 1996. Available from: <https://www.amazon.co.uk/Introduction-Quantitative-Genetics-Douglas-Falconer/dp/0582243025>

Malkin I, Ginsburg E. The Program Package MAN. Tel Aviv University technical report [Internet]. 2015. Available from: <http://www.tau.ac.il/~idak/hid_MAN.htm>

Malkin I, Williams FM, LaChance G, Spector T, MacGregor AJ, Livshits G. Low back and common widespread pain share common genetic determinants. Ann Hum Genet [Internet]. 2014;78(5):357–66. Available from: <https://www.ncbi.nlm.nih.gov/pubmed/24962672>

Supplmentary figures

Figure S1. FI scores distribution after root square transformation and standartization in study sample

**
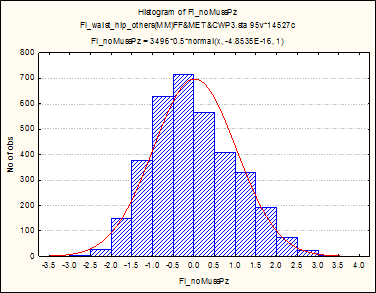
**

Figure S2. Age depednece of root square transformed and standartized FI scores in study sample
